# Exploring similarity patterns in a large scientific corpus

**DOI:** 10.1371/journal.pone.0321114

**Published:** 2025-04-21

**Authors:** Daniel Witschard, Ilir Jusufi, Kostiantyn Kucher, Andreas Kerren

**Affiliations:** 1 Department of Computer Science and Media Technology, Linnaeus University, Växjö, Sweden; 2 Department of Computer Science, Blekinge Institute of Technology, Karlskrona, Sweden; 3 Department of Science and Technology, Linköping University, Norrköping, Sweden; Educational Testing Service: ETS, UNITED STATES OFAMERICA

## Abstract

Similarity-based analysis is a common and intuitive tool for exploring large data sets. For instance, grouping data items by their level of similarity, regarding one or several chosen aspects, can reveal patterns and relations from the intrinsic structure of the data and thus provide important insights in the sense-making process. Existing analytical methods (such as clustering and dimensionality reduction) tend to target questions such as “Which objects are similar?”; but since they are not necessarily well-suited to answer questions such as “How does the result change if we change the similarity criteria?” or “How are the items linked together by the similarity relations?” they do not unlock the full potential of similarity-based analysis—and here we see a gap to fill. In this paper, we propose that the concept of similarity could be regarded as both: (1) a relation between items, and (2) a property in its own, with a specific distribution over the data set. Based on this approach, we developed an embedding-based computational pipeline together with a prototype visual analytics tool which allows the user to perform similarity-based exploration of a large set of scientific publications. To demonstrate the potential of our method, we present two different use cases, and we also discuss the strengths and limitations of our approach.

## Introduction

Within the fields of bibliometrics [1] and scientometrics [2], sensemaking of large textual corpora of publications is an important challenge, for instance when assessing important trends and topics within a specific research area [3,4]. One of the main goal of this task is usually to unveil the intrinsic structure of the data set in order to provide aggregated information about the content of the corpus (i.e., beyond the textual content of the contained articles). A common approach for revealing such structures is to exploit the similarity/closeness between the data items. Two prominent examples of such techniques are clustering [5] and dimensionality reduction (DR) [6]. These methods can provide important insights such as *“This corpus contain X clusters of similar documents”*, which in turn enables the user to build an elaborate mental model of the structural relations within the data set. However, while these methods are highly suitable for answering questions such as *“Which items are similar?”* [7], they are less well suited to answer questions such as *“How does the result change if we change the similarity criteria?”* and *“How are the items linked together by their similarity relations?”*. Since answering such questions may provide further important insights to the targeted corpus, we see that there is more potential to unlock regarding similarity-based analysis. In this paper we therefore propose a complementing methodology which focuses on the dynamic aspects of similarity criteria construction. This opens for new analysis approaches which, to the best of our knowledge, has not been explored in current research—and this is a gap that we aim to fill with our proposed contribution.

Similarity is an abstract and subjective relation which has been vigorously researched in the area of cognitive psychology [8–10]. The intrinsic subjectivity makes it challenging to evaluate similarity by a purely computational approach. A main reason for this is that the level of similarity between two objects hard to quantify in an objective way. On a more generic level, we note that the choice of relevant aspects to evaluate may differ from case to case (e.g., when designing an elevator, two humans can be considered similar if they have the same weight, but when designing clothes, height and gender probably also need to be considered). We can therefore think of the concept of similarity calculations as being specified by a dynamic set of similarity criteria which produce similarity links between the items of the data set. In other words, similarity X specified by the criteria *same eye color and weight* will produce a different set of similarity links over a population than similarity Y *same hair color and height*, since people who are similar when using criteria X may not be similar when using criteria Y. From this, we conclude that executing different similarity criteria over a data set will reveal different views of its intrinsic structure, each of which may provide important insights in the sense-making process.

**Fig 1:**
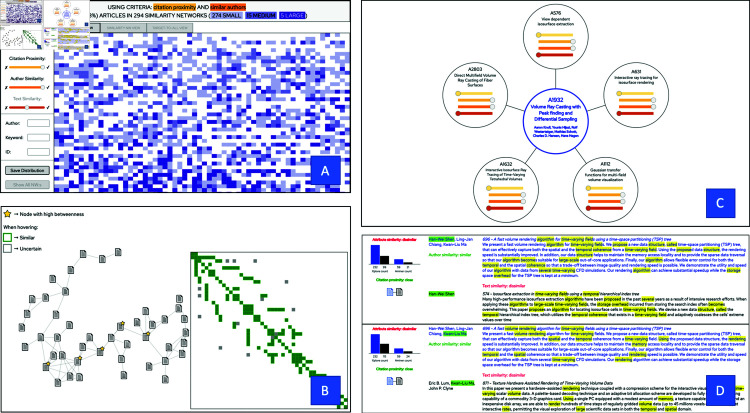
The user interface of Simbanex, a visual analytics tool for interactive similarity-based exploration of a large set of scientific publications. In the *Distribution View* [A], the distribution pattern of the current similarity criteria is displayed. In the *Similarity Network View* [B], the user can asses a selected similarity network and its corresponding adjacency matrix. The *Target-to-all View* [C] shows an overview of the matches for a selected article, and the detailed pairwise comparisons can be assessed in the *Similarity Assessment View* [D].

In this paper, we target the publicly available VISPUB [11] data set, which currently contains publications from the IEEE Visualization (IEEE VIS) conferences from 1990–2023 including IEEE TVCG and IEEE CG&A journal articles published at IEEE VIS. We have chosen this data set mainly due to our familiarity with the data; the analysis and exploration features discussed in this work are surely applicable to other scientific text corpora. We allow for four different aspects of the data to be included in (or excluded from) the similarity criteria: (1) abstract text, (2) author information, (3) numerical attributes, and (4) position in the citation network. Furthermore, we opt to use embedding technology for calculating the level of similarity for item pairs with regard to the different aspects. Embeddings are numeric vector representations of some underlying data, and they are normally produced in such a way that items which are similar in the original data set (according to some domain-specific aspect) are embedded into vectors that lie close to each other in the embedding space [12–14]. The rationale for choosing embedding technology is that it gives a uniform computational pipeline, and that the numeric vector format often can be more suitable than the original data as input for similarity-based computational analysis [15–18]. On this base we build a prototype visual analytics (VA [19]) application, called *Simbanex* see Fig 1), which is a further development of our previously proposed experimental tool *Simbatex* [20] (i.e., the tools are different but based on the same basic ideas). Simbanex is intended to be used within the fields of bibliometrics and scientometrics and allows the user to perform interactive similarity-based exploration of the underlying set of scientific documents. Our intended user is a scholar with only limited knowledge of machine learning technologies, aiming to explore the IEEE VIS corpus (in order to, for instance, gain better understanding of the corpus, or to write a survey on the content). The rationale for choosing this type of generic scope for the design of our tool, is that the functionality could be of value for many different analysis scenarios. We aim to provide a hopefully useful addition into the toolbox, rather than a rival to already existing methods. We therefore specifically want to point out that our proposed methodology and application are intended to be used in combination with other analysis methods, and that they therefore do not aim to cover the full scope of document exploration and mining. This specific context impedes traditional evaluation methods such as user studies with domain experts and comparisons to existing tools (since we do not target full-fledged analysis scenarios). Consequently, to demonstrate the usefulness of this type of similarity-driven exploration, we present two different proof-of-concept use cases, where the first focuses on citation link analysis and the second on topic similarity. The main contributions of our work are

1. a novel approach for similarity-based exploration of scientific publications;
2. a general methodology for multi-embedding similarity calculations, generalizable beyond the scope of bibliometrics/scientometrics;
3. an experimental VA tool, called Simbanex, which allows the user to interactively explore the similarity patterns of a large set of scientific documents; and
4. the presentation of two different use cases which illustrate the strengths of our proposed approach and showcase the usage of our tool.

The rest of this manuscript is organized as follows. First, we discuss the relevant related literature for this article. After that, the most important tasks and design goals are outlined. Next, we describe our computational pipeline. The specific details of our proposed VA tool are discussed afterwards, followed by the description of two different use cases. Finally, we conclude this article by discussing the outcomes, limitations, and future work stemming from our study.

## Related work

To set the context for our work, we start this section by outlining some of the key aspects from the fields of bibliometrics and scientometrics. We then continue with an overview of embedding technologies, where we focus specifically on those who we will use in our application.

### Bibliometrics and scientometrics

The concept of bibliometrics can be described as the application of mathematical and statistical methods to books, research papers, and other media [1], and within the subfield of scientometrics the focus lies on analyzing the quantitative aspects of scientific publications and their use. Ranking of publications and authors as well as generation of various aggregated statistical representations are common tasks, often in combination with visualization techniques to facilitate a better understanding of the underlying data [2]. So-called *distant reading* (i.e., using representations which convey information from the underlying text without the need for actually reading it) is an important concept that has been introduced to alleviate the inherent limitations of normal reading (which in turn is often referred to as *close reading*) [21]. Since close reading is time consuming, and time typically is a limiting factor, there is a high demand for distant reading applications which support the navigation of large document sets and convey relevant aggregated information, but still also allow on-demand access to the underlying text for detailed examination.

Natural language processing (NLP) in combination with visualization has proved to be a successful combination for tackling such challenges. Belinkov and Glass survey the impressive computational progress that has taken place in the field of NLP since the introduction of neural network models [22]. Kucher and Kerren [23] provide a taxonomy for, and an overview of, existing methods for text visualization. The survey of Federico et al. [24] focuses on visual approaches for analyzing scientific literature and patents while Liu et al. [25] target visualization and visual analysis of scholarly data, and Zhang et al. [26] focus specifically on visualization of topics in scientific literature. Finally, the BioVis Explorer by Kerren et al. [27] provides a way to navigate BioVis publications, and their connections, based on their respective visualization techniques.

Individual visual/interactive techniques and tools for supporting bibliometric/scientometric analyses often rely on the manually curated metadata available from the respective bibliographical databases, such as the DiVA Vis tool by Kucher and Kerren [28] for the Swedish DiVA university publication database or Argo Scholar by Li et al. [29] for Semantic Scholar data. Some other existing approaches rely not only on the metadata or citation analyses, but also topical analyses of the document contents, such as Cartolabe by Caillou et al. [30], which was primarily applied for the French HAL publication database. Interestingly, Caillou et al. mention the following when describing their methodology for HAL document text processing: *“we can use the full text or any section that would be the most informative, but due to the lack of quality measures to determine which sections of the text are the most informative ones, we use the simplest parts: the title, the keywords, and the abstract”* [30]. PUREsuggest by Beck [31] focuses on the search and foraging scenarios, such as creating and extending a literature collection based on the citation network analyses and user-defined keywords & rankings, while DocFlow by Qiu et al. [32] focuses on supporting systematic reviews of biomedical literature through question-based document retrieval based on a fine-tuned Transformer language model (see below). The InnovationInsights approach by Wang et al. [33] combines network analyses with further sources of information to support the exploration and prediction of research impact on innovation, such as technical patents. GlassViz by Benito-Santos and Therón [34] focuses on the challenge of finding commonalities across two disjoint sets of scientific publications through keyword analysis, including keyword embeddings. Finally, Lv et al. apply several dimensionality reduction techniques as well as topic analysis based on scientific publication embeddings to facilitate visual exploration of literature in materials science [35]. To summarize, the recent visual/interactive approaches are starting to combine metadata- and network-based analyses with document content analyses based on modern methods such as word and text embeddings, however, the body of work on the use of such approaches within bibliometric/scientometric analysis contexts is limited so far.

As can be noted from several of these publications, the scholarly domain in general, and the research domain in particular, are in themselves good examples of the bibliometric and scientometric challenges since the publication rate, in many research fields, makes it hard for any practitioner to maintain an overview and identify the most relevant information. A final observation that is relevant to our work is that it is not uncommon for corpus exploration to be in part driven by questions like *“Are there any groupings of similar documents within the set?”* or *“Are there documents which are similar to this specific document?”*. Therefore, the ability to exploit similarity relations is highly relevant for providing useful insights in the sense-making process of textual corpora [7].

### Embeddings

**Word and text embeddings.** In general, word embeddings are distributed representations obtained from training a deep learning model on some large corpus of natural language text [15,36–39]. The algorithms are trained on large amount of training data to predict words from a given a context, or the other way around. After training, the model can be used to compute word and phrase semantic similarities. The main idea is that the algorithm projects similar word pairs to embedding vectors that lie close to each other in the embedding space, so that semantic similarity can be calculated with the vectors as proxies [12,40]. One of the most influential word embedding algorithms is Word2Vec [41], introduced in 2013, and a current state-of-the-art model is BERT [42]. There are different approaches on how to use word embeddings to obtain embeddings for sentences or paragraph-sized text [43]. An intuitive method is to take the average of the embeddings of each word in the text, but to be able to exploit the syntactical structure of the sentences as well as phrasal semantics, more sophisticated approaches have been developed [44]. Exploiting the sentence structure is often necessary since the same word may have different meanings depending on the context, and the same set of words may be arranged to form sentences with very different meanings. For this, deep learning models are a popular choice, and approaches have, for example, been developed for recursive neural networks [45], convolutional neural networks [46], and recurrent neural networks [47]. Some of the most prominent recent approaches for embedding paragraph-sized text include the Universal Sentence Encoder (USE) [48] and the sentence version of the previously mentioned BERT model [49]. Recent large-scale computational benchmark comparisons of multiple text embedding methods showed that no single method dominated across all of the respective tasks [50]. Furthermore, the advances in large language models (LLMs) over the past several years [51] have recently led to the attempts to produce or improve text embeddings by leveraging LLM capabilities, including the efforts from research teams from Microsoft [52] and Meta/Facebook [53].

**Graph and network embeddings.** Embedding calculations are not exclusive to textual data, for instance, they can be applied to various important tasks and applications involving graph and network data [54,55]. Technology for graph embedding, also known as Representation Learning on Graphs [56], targets the pure topological structure of the graph. The goal is to preserve as much as possible of the structure information and important tasks are clustering, graph comparison, and graph reconstruction. Depending on the application, the item(s) to embed may be: (1) the whole graph, (2) subgraphs, (3) the nodes, or (4) the edges [13,57]. Furthermore, even dynamic aspects can be taken into account for embedding purposes [58]. The field of network embedding [59] is closely related to the field of graph embedding. The main difference is that in addition to the graph topology some (or all) of the attributed data is also considered, which allows for a more elaborated embedding process. Consequently, this type of technology is sometimes referred to as Attribute Enhanced Representation Learning [14].

## Analytical tasks and design goals

In this section, we outline the most important analytical tasks and the most important design goals that were set out for the development of the Simbanex tool. We would like to start by pointing out that Simbanex is a prototype visual analytics tool which is intended to showcase the potential of similarity-based analysis, rather that showcasing full-fledged analysis scenarios within the field of bibliometrics. This in turn, means that the tasks and goals listed in this section are not necessarily intended to cover a full analytical pipeline.

Two analytical tasks were specifically targeted as specified below. These tasks were selected by the criteria that they demonstrate real-world value, and can be performed by using similarity-based analysis. Both of these tasks are outlined as detailed use cases in the respective section. Of course, these are not the only tasks that could be solved (in full or partially) by using Simbanex.

*[T1] Citation pattern analysis (Use case 1).* Exploiting the citation links of the publications in order to infer general patterns that could give insights to the structural relations within the corpus.
*[T2] Topic similarity analysis (Use case 2).* Exploiting the semantic similarity of the publications in order to infer groupings of similar articles and/or topic clusters.

The specific design goals which were set out are listed below. In Sect “Visualization approach”, we will return to these goals and specify how they were fulfilled.

**D1** To invite the user to reflect on the subjective nature of similarity.
**D2** To showcase some specific similarity-based analysis scenarios/capabilities.
**D3** To present the data by providing a meaningful visual schema.
**D4** To provide useful interactive capabilities to help investigate the data.
**D5** To facilitate the trust-building process by helping the user to build a mental model of the inner workings of the similarity calculations.

## Computational approach

**Fig 2:**
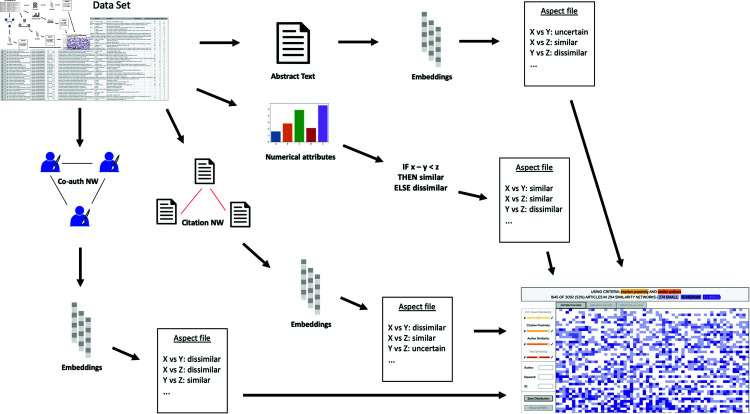
A schematic view of how the four different aspects have been extracted from the original data. For each aspect, the pairwise similarity classifications (i.e., *similar*, *dissimilar*, or *uncertain*) are calculated, and loaded into the application. This allows for constructing flexible dynamic queries, and handling various analysis scenarios, in Simbanex (see Sects “Computational approach” and “Visualization approach”). This methodology can be generalized to any data entity which may be divided into separately embeddable aspects.

In this section, we outline and discuss the major steps that are used within the computational pipeline of Simbanex. The main idea is to use our previously developed methodology for multiple-embedding similarity calculations [60] to determine several different aspect-specific classifications using the three classes *similar*, *dissimilar*, and *uncertain*. These classifications will then form the base for the similarity-based analysis in the application (see Fig 2). A coarse grained list of the process is given next:

1. Divide the data into several different aspects.
2. Embed each aspect separately.
3. Determine the aspect classifications.

To better illustrate the above, we will now go through how each step was applied to our data set. We use the IEEE VIS data set [11] which contains information of articles published at the IEEE VIS conferences. The choice of the data set is motivated by its quality, visibility in our research community, and our own familiarity with its topic. From this set, we have extracted approximatively 3,000 articles from which we have built an attributed citation network where each node corresponds to a publication and holds the following data: (1) the abstract text, (2) the co-author information, and (3) two different citation counts (IEEE XPlore and AMiner).

### Step 1—Divide into aspects

Apart from actual text from the publications, the citation network [61] and the co-author network [62] have proven to be important aspects of the underlying corpus when it comes to document mining tasks in bibliometrics [2]. Therefore, in our implementation, we use the following aspects:

1. The abstract text
2. The co-author information
3. The citation network topology
4. The citation counts

The citation counts have been included to allow for similarity comparisons of impact (e.g., publications with high citation counts are similar in the sense that they have had a high impact on their respective fields). We include self-citations in both the citation counts and the citation network.

### Step 2—Handle each aspect

For each publication, we now use existing state-of-the-art embedding methods to create several different embeddings for each aspect as described in the following:

**Abstract text**

We use paragraph text embedding technology to embed the abstract text of the publications. The assumption is therefore that the embedding vectors of abstracts that are semantically similar to each other will yield a high cosine similarity score, and that the vectors of abstracts that are semantically dissimilar will yield a low cosine similarity score. In our computational pipeline, we can use Sentence-BERT [49], USE [48], SPECTER [63], or LLM2Vec [53] with the Meta-Llama-3-8B model [64] for this step. We obtain five different embedding vectors per publication by feeding the chosen model five different variants of the text as follows:

*V1* – Embed the whole abstract text and thus capture overall similarity.
*V2* – Embed the first 400 characters of the abstract text and thus capture similar beginnings, but ignore everything else.
*V3* – Embed the last 400 characters of the abstract text and thus capture similar endings, but ignore everything else.
*V4* – Concatenation of V2 and V3. Capture abstracts with similar beginnings and similar endings.
*V5* – Embed keyword sentences extracted from the abstract text and thus capture overall similarity, but with the risk of the keywords not being representative. The keyword extraction is made with the RAKE algorithm [65].

The limit of 400 characters has been set in relation to the average length of the abstracts, which is just below 1,000 characters. The rationale for using not only the full abstract text is that having several different embeddings of the data will enable a better classification of borderline cases (i.e., we will, loosely speaking, have several different “opinions” on the level of similarity that we can combine to a final answer). Details of such combination strategies are presented in our previous work with a different focus [60] and fall outside of the scope of this paper. The choice of the BERT, USE, and SPECTER models is based on the fact that they are all state-of-the-art approaches for text embedding, and therefore they should be able to adequately capture the similarity distribution over a document set. The addition of LLM2Vec [53] gives the possibility to leverage the rapid advances in the field of large language models (LLMs) [51]. Detailed text embedding model comparisons also fall outside the scope of this paper (to that end, we refer the readers to the recent MTEB benchmark study by Muennighoff et al. [50]), however, we note that using the LLM approach yields no obvious difference in quality as compared to the other three models in our case. The rationale for not choosing more than four models is that we are aiming to develop a new methodology rather than analyzing which model that performs best, and for this purpose four models are enough. However, based on the progress in NLP [50,51], models could easily be added or exchanged for others.

**Co-author information**

We use neighbourhood-aware embedding technology to embed the nodes of the co-author network that is associated with our corpus (i.e., nodes that lie close within the network will be embedded into vectors/points that lie close within the embedding space). To handle the fact that there are usually several co-authors of a publication, we take the average of all the corresponding author node embedding vectors (which is a valid approach since the neighborhood-aware approach ensures that co-authors have been embedded close to each other). Hence, the resulting single embedding captures information of all co-authors, and it yields high cosine similarity scores from vectors of articles that have co-occurring authors. We use three different algorithms for this step (RandNE [66], Node2Vec [57], and Laplacian Eigenmaps [67]), and hence we obtain three different embedding vectors per publication regarding this aspect. The choice of these three models is based on the fact that they are based on very different computational approaches, and therefore together they should be able to complement each other in capturing the similarity distribution over a document set. Details of the individual model performances are presented in another previous publication [68] and also fall outside of the scope of this paper. Also in this case, the rationale for not choosing more models is that we are aiming to develop a new methodology rather than analyzing network embedding model performance, and the models are easily interchangeable in our computational pipeline.

**Citation network topology**

We use neighbourhood-aware technology also to embed the nodes of the citation network that is associated with our corpus. Therefore, the process steps are quite similar to the steps for the co-author information (with the difference that no averaging is needed). Generally speaking, the algorithms will exploit the network topology to capture structural aspects and produce embedding vectors for each of the nodes/articles within the citation network. These embedding can then be directly used for similarity calculations regarding the article nodes. In this case, the assumption is that the embedding vectors will yield a high cosine similarity score for article nodes that lie close to each other and/or belongs to the same citation context. We use the same three algorithms as for the co-author network and therefore obtain three different embedding vectors per publication also for this aspect.

**Citation counts** For the two citation counts we directly calculate the similarity classification by applying the following rules:

1. For counts below 100, a maximum difference of 10 is allowed. (E.g., 2 and 8 will be regarded as similar, but 5 and 17 will be regarded as dissimilar.)
2. For counts between 100 and 500, a maximum difference of 50 is allowed. (E.g., 122 and 161 will be regarded as similar, but 328 and 381 will be regarded as dissimilar.)
3. For counts above 500 a maximum difference of 500 is allowed. (E.g., 537 and 968 will be regarded as similar, but 1,044 and 1,613 will be regarded as dissimilar.)

**Fig 3:**
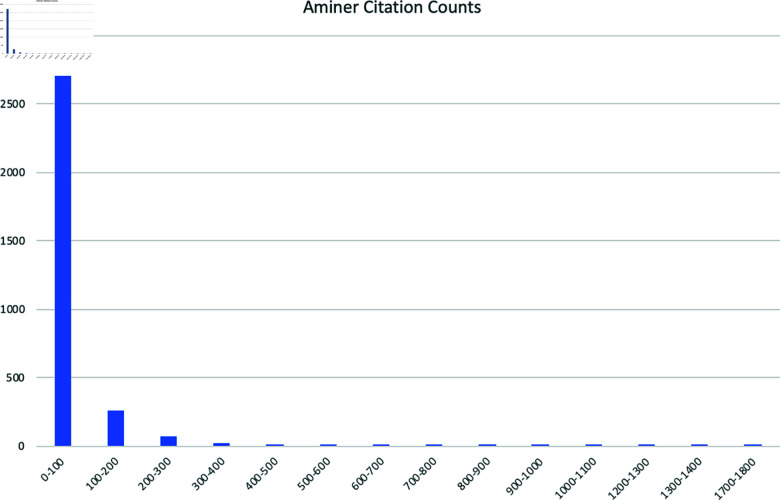
The AMiner citation counts represented as a histogram with bin size 100. As can be seen, the bulk of the data is heavily skewed to the left with a long tail to the right. From this distribution, it is reasonable to argue for the use of different “similarity binning granularity” depending on location on the x-axis. Using only a large bin would make all of the observations to the left similar, and using only a small bin would make no observations to the right similar.

The rationale for the above set of rules is that it is not uncommon to have different “similarity binning granularity” depending on where on the scale we are currently measuring. For example, many people would most likely regard a citation count of 2 to be quite different from a count of 53, but at the same time regard a citation count of 438 to be quite similar to 502, although the distance between the two former is less than between the two latter. The specific bins have been determined by assessing the histograms of the citation counts of the data set (see further Fig 3).

### Step 3—Determine aspect classifications

As specified above, two different similarity classifications for the citation counts have already been calculated (one for AMiner and one for IEEE XPlore. To determine the classifications of the other three aspects we first calculate the pairwise cosine similarity scores for all embedding types. As specified in Step 2, a total of 11 different embedding vectors have been created per publication (5 for the abstract text, 3 for the author information, and 3 for the closeness in the citation network). Consequently, for each article pair we can now calculate 11 different similarity scores and group them by their corresponding aspect. By using the methodology developed in [60] we search for the best possible threshold scores for determining whether two articles should be connected by a similarity link or not (i.e., item pairs with similarity scores equal to or above the threshold are classified as similar and pairs with scores below the threshold are classified as dissimilar). Low threshold scores will produce many similarity links, but possibly of questionable quality, while high threshold scores will produce links of high quality, but possibly in low quantity. The process should ideally make use of some a priori ground-truth knowledge, so that the quality of the yielded result can be evaluated, and finding the best possible settings is a balance between quality and quantity (a more detailed discussion of this process falls outside the scope of this article). Since all models yield a different distribution of the pairwise similarity scores (e.g., some models yield, in general, higher scores than others) the model-specific thresholds are not directly comparable to each other on their numerical value. The threshold settings used within our implementation are specified in Tables 1 and 2. However, it is important to underline that: (1) there is no truly objective way to find an optimal threshold, and therefore (2) choosing other thresholds is perfectly viable and would change the result in the sense that the number of pairs classified as similar will be different from setting to setting. We then derive the pairwise aspect classifications as follows:

**Table 1:** Threshold scores for the text embeddings. The threshold scores have been set by using the methodology developed in our prior work [60]. There may be a considerable difference in the distribution of the pairwise similarity scores across models (e.g., 0.85 may be a relatively high similarity score from one model, but a relatively low score from another), so the numerical values cannot be directly compared across models.

	USE	BERT	SPECTER	LLM
Full	0.84	0.87	0.95	0.89
First	0.77	0.87	0.96	0.86
Last	0.70	0.85	0.93	0.84
First & Last	0.67	0.80	0.93	0.80
Keywords	0.75	0.83	0.94	0.80

**Table 2:** Threshold scores for the network embeddings. The threshold scores have been set by using the methodology developed in our prior work [60]. Also for these models there is a considerable difference in the distribution of the pairwise similarity scores.

	RandNE	Nod2Vec	L. Eigenmaps
Co-author	0.36	0.59	0.75
Citation	0.38	0.63	0.74

1. We classify a pair as *similar* with regard to an aspect if a majority of the aspect specific similarity scores “vote” for this.
2. We classify a pair as *dissimilar* with regard to an aspect if a majority of the aspect specific similarity scores “vote” for this.
3. If none of the previous holds true, we classify the pair as *uncertain* with regard to this aspect.

This step clearly shows the benefit of having several similarity scores for each aspect since it allows for the use of a combiner function (in this case voting) to leverage the single-score classifications and augment the quality of the result. Hence, at the end of this step, each article pair has obtained 4 classifications, specifying how similar the two publications are with regard to each of the aspects, and these results are now ready to be loaded into Simbanex.

### Scalability

Regarding the scaling potential of our approach, we note that some embedding algorithms allow for asynchronous case-by-case embedding (e.g., USE and BERT who are pre-trained), while others (such as RandNE and Node2Vec) rely on embedding all data at once. Embedding the data is the computationally most demanding step of our pipeline, and therefore our approach is better suited for scenarios where all data is collected up front rather than arriving sequentially. We also note that there is an inherent scaling limitation for using pairwise strategies on large data sets, since the number of pairs grows with the square of the number of items. Taking all of this into consideration, our experiments show that our proposed approach is adequate for analyzing corpora up to the size of 10,000 documents, which makes it viable for several real-world use cases. Scaling the computational and visual/interactive components of our approach for data sets of considerably larger magnitudes can be considered part of future work [69], for instance, by relying on progressive data analytic methods [70].

## Visualization approach

In this section, we give an overview of the visual design of Simbanex, which is implemented as a web-based tool using the D3 JavaScript data visualization library [71]. The overall aim of the tool is to fulfil design goal **D1** by allowing the user to dynamically explore the distribution of different similarity criteria over the data set, with drill-down functionality so that the analysis can be performed at different levels of detail. Furthermore, in order to fulfill design goal **D3**, the overall design seeks to reuse already well-proven visual metaphors whenever possible. As can be seen in Fig 1A, Simbanex consists of a control panel (to the left), a banner for context-dependent textual information (top), and a main area for displaying results.

In the control panel, there are four sliders for the similarity criteria (*Citation Count Similarity*, *Citation Proximity*, *Author Similarity*, and *Text Similarity*). By adjusting the slider positions the user may dynamically construct different similarity criteria and assess the yielded results. The sliders have three positions and allow the user to select NO (left position), DISABLED (middle position), or YES (right position) for each individual criterion. Setting a slider to YES means *“Find all pairs that are classified as similar for this aspect”*, setting a slider to NO means *“Find all pairs that are classified as dissimilar for this aspect”*, and setting the slider to the middle, disabled position means *“Do not use this aspect for filtering purposes”* (see further Sect “The distribution view”). There are also fields for which allow the user to search for publications based on author, keyword, or article ID. Matching articles will be highlighted throughout the views (see further Sects “Tracking” and “Use case 2 – Topic similarity”). And finally, there are interaction buttons as explained below (see also Sect “Use cases”). The design of the control panel is intended to fulfill design goals **D1** and **D4**.

*Save Distribution* Enables the user to save snapshots of the content in the distribution view so that the yield of several settings can be compared to each other. When snapshots have been saved, the *SAVED DISTRIBUTIONS* tab will appear in the main area and contain an indicator showing the number of saved items. *Show All NW:s* This button is enabled when it is possible to display all the currently constructed similarity networks together (see Sect “The similarity network view” for details on similarity network construction). *Clear Selections* Deletes all saved snapshots and resets all settings to the initial state. *Upload Abstract* Enables the user to upload an abstract and compare it to the articles in the data set (see Sect “Use case 1—Citation link analysis” Step 6 for details).

**Fig 4:**
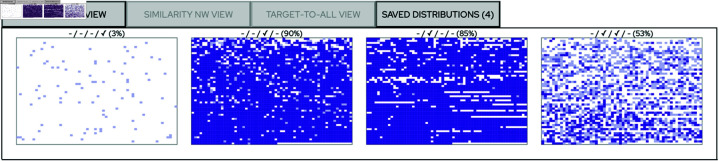
Comparing different distributions as part of the sense-making process. In this example the user has saved and displayed the distributions of 4 different criteria settings. The low color intensity in the leftmost miniature indicates that text similarity is uncommon within the corpus, and the light hues indicate that the similarity networks that can be constructed from this aspect are small. In contrast, the high color intensity and dark hues indicate that author similarity (middle left) as well as citation proximity (middle right) are common and yield large similarity networks. Furthermore, the combination of author similarity and citation proximity (rightmost) affects approximately half of the articles and yield similarity networks of mixed sizes. Each of these views therefore convey a different view on the intrinsic structure of the corpus that may be important in the sense-making process. The information above the miniature charts indicate the amount of affected articles and which slider settings that were used, and it should be read as: *Slider 1 position / Slider 2 position / ...* where “-” indicates that the slider was disabled.

To avoid too small and/or too cluttered displays, we have chosen to utilize the main area (after clearing it from previous information) also for displaying details when drilling down into the data. This is accomplished by providing four main views, each at its own level of abstraction: (1) the *Distribution View* (see Fig 1A), (2) the *Similarity Network View* (see Fig 1B), (3) the *Target-to-All View* (see Fig 1C), and (4) the *Similarity Assessment View* (see Fig 1D). The combination of these four views, together with the interaction capabilities, fulfilling design goals **D2** and **D5**. The first three of these views are accessible (when populated) by the three tab buttons at the top of the application, and the *Similarity Assessment View* is displayed in combination with the *Target-to-All View*. Furthermore, the navigation between the views (apart from using the tab buttons) is accomplished in the following way: (1) clicking a colored rectangle in the *Distribution View* will display the *Similarity Network View* for the similarity network that the article belongs to, and (2) clicking an article icon in the *Similarity Network View* will display the *Target-to-All View* for the selected article. To facilitate the interaction and to clarify provenance, the banner continuously displays contextual information of the settings and the results to the user.

### The distribution view

When the visualization is loaded, the publications are represented by article icons in the *Distribution View* sorted on row number (as from the CSV-file of the data set) left to right and top to bottom (see Fig 1A. This, in turn, gives a chronological structure of the display since the CSV-file is ordered by conference year, oldest first. When the user selects a combination of slider positions, the corresponding similarity criteria will be executed over the set of publications. Article pair which are classified as similar will be connected by similarity links, and when the process has finished, a set of similarity networks will thus have been constructed. The distribution view will display information regarding the constructed similarity networks by replacing the article icons with a colored rectangle in the following way: (1) an article which does not belong to a similarity network (i.e, it does not have any similarity links to other articles) will be replaced by a white rectangle (and thus disappear from the display). (2) An article which belongs to a similarity network will be represented by a colored rectangle, where the color intensity corresponds to the size of the similarity network (the larger the network, the darker the color). The result yielded by the chosen similarity criteria is hence presented to the user as a colored pattern (see Fig 2 bottom right corner and Fig 4). The pattern conveys information on the number of affected articles as well as the sizes of the constructed similarity networks, and the goal of the design is that these properties should be easily distinguishable and comparable.

**Fig 5:**
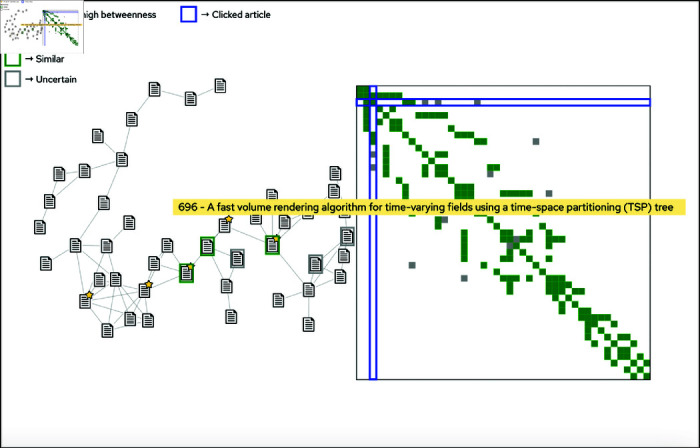
The *Similarity Network View.* Clicking a colored rectangle in the *Distribution View* displays the similarity network that the article belongs to, both in a node-link diagram (left) and in an adjacency matrix (right). As can be seen, similarity is not necessarily a transitive relation, so while the items of the network are all connected by at least one path they are still not necessarily all similar to each other. The network topology reveals the transitive properties of the selected similarity criteria, and can be used to establish an indirect similarity-path between two objects that are not similar when directly compared. In this example, the network topology reveals both more densely connected groupings of documents as well as chains of documents. We can also see documents who act like bridges between groupings and therefore are highlighted by a star. The user is hovering the mouse cursor over an article icon to highlight matches, as well as the node’s position in the adjacency matrix.

Finally, we need to point out the fact that it is not possible to build similarity networks if no slider is put in the YES position. Therefore, if all activated sliders are in the NO position, Simbanex will use different colors and a different banner message to make this clear to the user.

### The similarity network view

Clicking a colored rectangle in the *Distribution View* displays the *Similarity Network View* (see Fig 1B and Fig 5) where the similarity links between the articles are displayed, both in a node-link diagram and in an adjacency matrix. The purpose of the view is to visualize how the documents are linked together by the current similarity criteria (i.e., a link between two documents indicate that they have been classified as similar). This is important since it gives the analyst a clearer view of the similarity relations within the data. The network topology reveals information on the transitive property of the selected criteria, as well as on the overall pairwise homogeneity/heterogeneity of the nodes within the network. This in turn allows for nuanced similarity analysis, since two items that are not similar when directly compared may still be connected by an indirect “similarity-path”, and may therefore still be similar to some extent. Furthermore, the network topology can also be used to find items which act as bridges between groups of items with higher inter-connectivity. Since such items can be important to locate and analyze further, Simbanex highlights nodes with high betweenness centrality with a golden star. As previously discussed, depending on the selected criteria combination different similarity networks may be constructed for the same set of items. For example, when using one set of criteria we might get the result *“X and Y are similar AND Y and Z are similar BUT X and Z are dissimilar”*, and when using another set of criteria we might get the result *“X and Y and Z are all similar”*. When the user hovers an article node, the application highlights its similarity matches, as well as the corresponding row/column of the adjacency matrix. As can be seen in Fig 5, although the node-link diagram (left) and the adjacency matrix contain the same information (i.e., how the nodes are linked together), they layout/convey this information differently and can therefore complement each other as analysis tools. For instance, depending on the topology of the network, groupings and chains of documents can be more easily distinguishable in one layout than the other.

**Fig 6:**
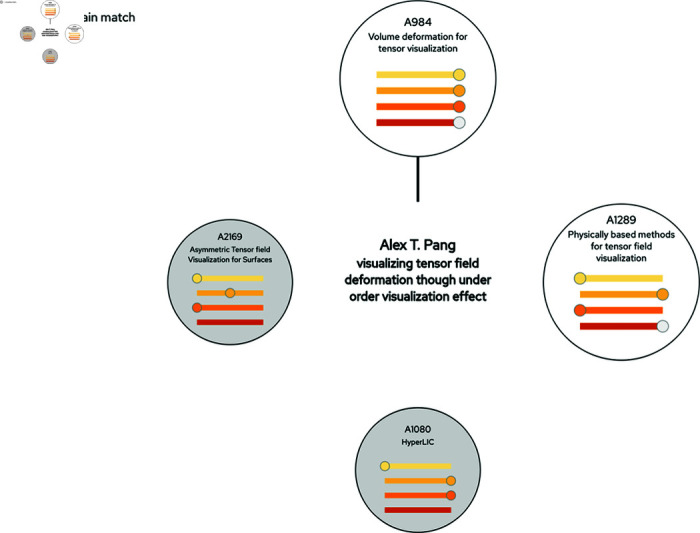
Clicking an article icon in a similarity network displays the radial *Target-to-All View.* The design of this view is intended to facilitate an at-a-glance assessment of all matches in the data set for the selected target. The colored charts inside the nodes are miniatures of the sliders. Activated slider settings are indicated with a grey slider button, and for non-activated sliders the button is in the same color as the slider background, and its position indicates which setting that would result in a match for the corresponding aspect. For uncertain matches, there are no slider buttons on the corresponding sliders. In this example, the user has put the *Text Similarity* slider to YES, and has clicked an article in a similarity network, which has resulted in 2 matches (white circles) and 2 uncertain matches (grey circles). The user is hovering the comparison node for article 984 (the upmost circle) to display co-occurring authors and co-occurring words. The analysis of these matches reveal that these four articles treat the same topic, i.e., “tensor field visualization”. Extending the analysis by selecting one of the matches as the target can reveal if there are more documents belonging to this topic grouping, or if the new target also simultaneously belongs to other topic groupings.

### The target-to-all view

Clicking an article icon in the *Similarity Network View* displays the *Target-to-All View* (see Fig 1C and Fig 6). In contrast to the Similarity Network View, which focuses on general patterns within the data, the purpose of this view is to focus on how similar a selected target article is to any of the other articles, given the current criteria settings. This allows for a more detailed analysis of documents of specific interest, for instance when analyzing if a grouping of articles treat the same topic. The selected article (i.e, the target) is represented by the node in the middle , and all matches are displayed in a radial layout which aims to provide an efficient at-a-glance overview of the pairwise comparison of each aspect. The chart offers information on the level of similarity also for currently disabled criteria sliders. Furthermore, by hovering a comparison node, the user can display co-occurring authors and co-occurring words for the central article and the hovered one. In Fig 6, an example is shown where a selected article has links to two articles that have been classified as similar, and it also has links to two others where the classification has been set to “uncertain”.

### The similarity assessment view

The *Similarity Assessment View* (see Fig 1D) is displayed just below the *Target-to-All View* and shows the full details of all of the pairwise comparisons. To facilitate the assessment, the data for the selected target article is color-coded in blue, and co-occurrences of words and authors are highlighted with colored spans. Furthermore, all four system-generated aspect classifications are displayed so that the user can assess them in the direct context of the actual data. This view allows the user to form his own opinion on the level of similarity of the article pair, and to compare it with the results of the computational analysis.

### Tracking

To allow for more specific search and analysis scenarios, the user may track articles on author name(s) and/or keyword(s) by using the provided search fields. Doing so triggers Simbanex to highlight matching articles with blue-colored frames throughout the views. The tracking feature is helpful for answering questions such as: *“Do articles that mention certain keywords also show high overall similarity?”* among others (see further Sect “Use case 2—Topic similarity”).

### Comparison to other tools

As previously stated, Simbanex is a prototype VA tool which allows the user to perform interactive similarity-based exploration of the underlying set of scientific documents. The rationale for choosing this type of generic scope for the design of our tool, is that the functionality could be of value for many different analysis scenarios. Consequently, our aim is to provide a useful addition to the toolbox, rather than a rival to already existing methods. We therefore specifically want to point out that our proposed methodology is intended to be used in combination with other analysis methods, and that Simbanex does not aim to cover the full scope of document exploration and mining. Therefore, Simbanex cannot be adequately compared to analysis solutions for scientific corpora such as VOSviewer [72,73] or SciMAT [74,75] since they have been developed with broad full-fledged biblometric/scientometric analysis scenarios in mind. Extending Simbanex to cover complete analysis workflows (for instance from the design spaces discussed in the surveys by Federico et al. [24] and Liu et al. [25]), can be considered part of future work.

## Use cases

We explore a novel and specialized application domain and our proposed application only targets a subset of normal corpus mining functionality, and traditional evaluation methods (such as user studies with domain experts or comparisons to existing tools) are therefore not a viable option. In this section, we instead outline two different use cases that highlight some of the strengths of the similarity-based approach. These two use cases have been selected to showcase how the methodology could be used as a part of realistic applications in bibliometrics and scientometrics [24,76,77].

### Use case 1—Citation link analysis

Simbanex makes it easy to interactively explore and get a better understanding of some of the citation patterns within the set of publications (our analytical task **T1**). The similarity-based approach makes it possible to distinguish between citations between publications with similar abstracts and citations between publications with dissimilar abstracts, and this can be exploited for different tasks.

**Fig 7:**
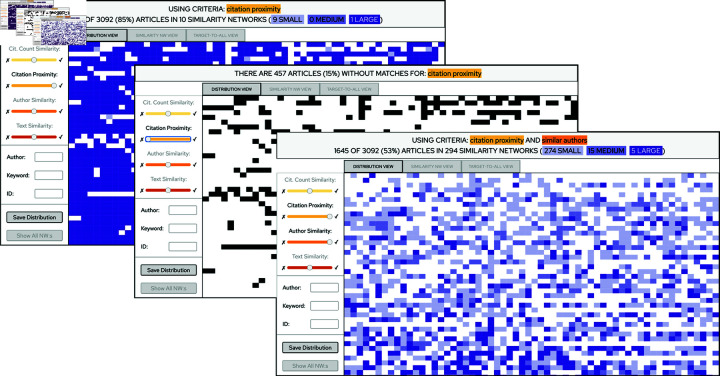
In the first three steps of Use Case 1 (in order from the background to the fore), the user first explores the general citation pattern, and then the level of self-citation.

**Fig 8:**
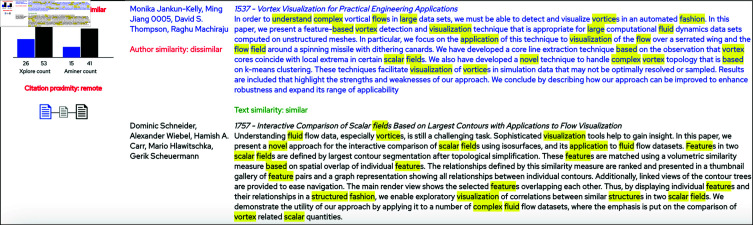
One of the suggestions for “missing citations” from Use Case 1. As can be seen, there is already an indirect (1–hop) link between the two publications, and maybe it could have been relevant to have a direct citation link between the articles instead. The common words of the abstracts are highlighted in yellow.

**Fig 9:**
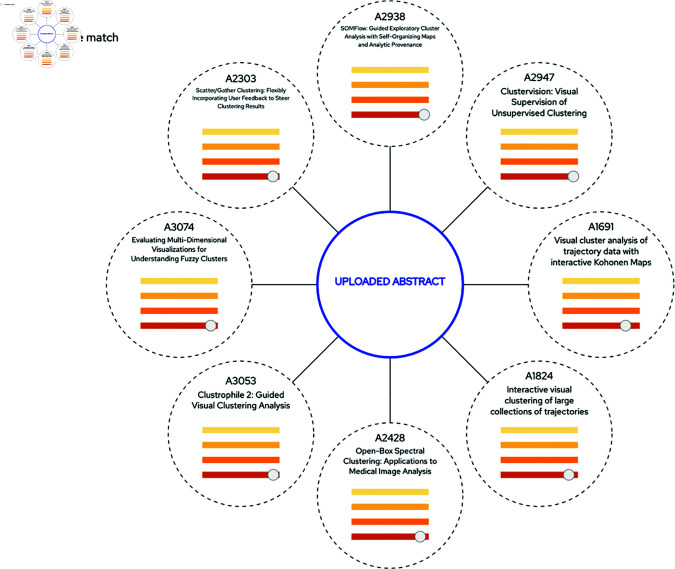
When an abstract is uploaded, the system displays possible matches (if any) based on text similarity. The slider button position indicates the level of similarity (the higher the similarity, the more to the right the button is positioned. The user can then inspect each match individually in the *Similarity Assessment View* to verify if it makes for a relevant citation or not.

1. Starting with the simple case of determining the overall citation level, the user puts the Citation Proximity slider to YES and can quickly assess that roughly 85% of all publications have citation links to another publication within the corpus. From the *Distribution View* he can also conclude that the vast majority of these articles belong to one single connected component of the citation network, see Fig 7 (background).
2. Switching the Citation Proximity slider to NO makes it possible to assess the other 15% of the publications that do not cite publications within the data set (a so called *outgoing* citation link) and are not cited by any other publication within the data set (a so called *incoming* citation link), see Fig 7 (middle). The displayed pattern has more color intensity in the upper part of the view. Since the view has a chronological structure from top to bottom (with the oldest publications at the top, see Sect “The distribution view”), the user can infer that an unproportionally large amount of these cases are from early years with regard to the time span of the data set. He concludes that this is to be expected since this means that these publications would have less previous articles to cite within the data set to cite which will substantially lower their probability for having an outgoing citation link. Interestingly enough, very few publications from later years of the time span are found within the subset even though the reverse effect (i.e., a lower probability for incoming citation links), would be expected for these articles. The user therefore concludes that citing within the data set is a trend that has grown stronger over the years and that it is very common for recent publications to do so.
3. To assess the level of self-citation, the user now sets both the Citation Proximity and the Author Similarity sliders to YES and concludes that the self-citation links exist for about 53% of the publications. This time the pattern in the *Distribution View* indicates that these criteria result in many similarity networks of various sizes, see Fig 7 (foreground).
4. The user then decides to explore whether he can find any “missing” citation links between similar publications, and therefore sets the Citation Proximity slider to NO and the Text Similarity slider to YES. This reveals that there are 11 article pairs with high text similarity and no citation link. Flipping the Author Similarity slider shows that 8 of these pairs have high pairwise author similarity, and that 3 pairs do not share any common authors.
5. The user focuses on the 3 pairs with low author similarity and clicks the *Show All NW:s* button. He then clicks on the article nodes to assess the detailed pairwise similarity to verify if the matches qualifies as possible citations that should have been made, see Fig 8.
6. Conscious about the previous results, the user plans to make a submission to an upcoming IEEE VIS conference and does not want to miss to cite any previously published articles with similar content. He therefore creates a set of embedding files (as specified in Sect “Step 2—Handle each aspect”) for his abstract and saves the result into a specified directory of the application (in the current implementation we only allow for embedding with USE for this functionality). He then selects the *Upload Abstract* button to display the *Target-to-All View* which is now centered around his uploaded text. The radial nodes consist of articles with high semantic text similarity (if any) and the user may use this view and the *Similarity Assessment View* to make an assessment of whether they are relevant candidates for citation or not, see Fig 9.

### Use case 2—Topic similarity

For the second use case, we will use Simbanex to locate sub topics within the context of selected keywords (our analytical task **T2**). Note that within this section we use the term “topic” in a loose sense (i.e., a subject that is treated by several publications,) which is not totally equivalent to the meaning that is implied within more formal methods of topic modeling.

**Fig 10:**
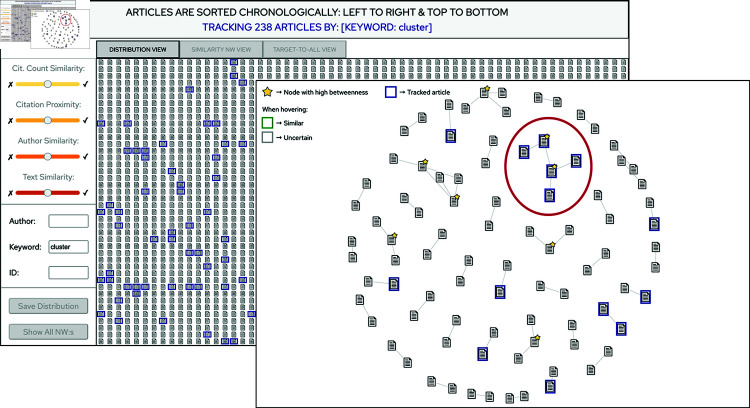
When tracking on keywords and/or author names the corresponding articles are highlighted in blue frames throughout the views. In this example from Use Case 2 (see Sect “Use case 2—Topic similarity”), the user observes a similarity network where all 5 publications mention the word “cluster” (the network highlighted by the added red ellipse). Further assessment of the abstract texts shows that they form a topic cluster on “visual cluster analysis”.

1. In this use case, another user has a special interest in finding out if there are any specific sub groupings within the set of publications for which clustering is an important topic. She therefore enters the keyword “cluster” into the keyword search field and gets a result of a total of 238 articles (which are now highlighted with blue frames), see Fig 10 (background).
2. Since it is still not an easy task to assess whether these 238 articles form smaller topic clusters or not (within the larger scope of clustering), the user sets the Text Similarity slider to YES, which triggers Simbanex to construct similarity networks and calculate the distribution over the data set.
3. From the pattern in the *Distribution View*, she concludes that only a small fraction of the tracked articles are among the article pairs which are linked together by high semantic similarity, which at first seems discouraging.
4. To further investigate, the user now clicks the *Show All NW:s* button to display the current similarity networks, and in the *Similarity Network View* she notices a network where all 5 articles have blue frames, see Fig 10 (foreground).
5. By clicking the article nodes of this network the user can display the *Target-to-all View* to compare the abstract texts. When doing so, the user observes that the articles all treat a subtopic that could loosely be described as “visual cluster analysis”. Furthermore, for some of the articles there are also uncertain matches of which one is relevant, so the article set can be expanded further to a total of 6 articles.
6. By noting that the discovered subset of articles would not have been easily distinguished only by a combination of keywords, or by traditional topic modeling, the user concludes that there are cases when similarity-based analysis can be a good method for topic detection.

## Discussion and conclusions

In this paper, we have outlined a novel approach for similarity-based analysis, and also presented a VA solution to address this analytical challenge. In this section, we discuss the strengths and weaknesses of our approach from various perspectives.

### Novel approach

By proposing a methodology which allows for dynamic construction of similarity criteria, we have shown how more potential can be unlocked in similarity-based analysis. Existing analytical methods (such as clustering and dimensionality reduction), tend to focus on questions such as *“Which items are similar?”*, and our method complements this approach by providing a more dynamic view on what similarity is, and how it can be specified. With its intended use within the fields of bibliometrics and scientometrics, we have shown how our proposed similarity-based approach can be used to obtain a better understanding of important aspects of the underlying set of scientific publications (beyond the textual content of the participating articles). As a proof-of-concept, we provide two different use-cases that showcase how Simbanex’ ability to dynamically specify similarity criteria enables the user to obtain several insights on the intrinsic structure of the corpus that would not have been easily obtained by other methods. Furthermore, one main advantages of our computational pipeline, is that it provides a homogeneous framework for the similarity calculations, and that the methodology generalizes to any complex data type that may be broken down into separate aspects. Finally, since similarity is an elusive and almost philosophical concept (that very much lies in the eye of the beholder), it is important to stress the fact that a purely computational approach is not sufficient and that a human-in-the-loop solution is needed.

### Limitations and future work

Our choice of using embedding technology for the similarity calculations also comes with some disadvantages, since it makes the scalability of our solution rely on the characteristics of the chosen embedding algorithms. While some algorithms allow for asynchronous case-by-case embedding (e.g., USE and BERT who are pre-trained), many others rely on embedding all data at once (i.e., a total recalculation is needed if data is added). However, our main idea is not dependent on how the aspect similarity is calculated, so (at the cost of lost homogeneity in the computational pipeline) other method(s) could be used instead. We also note that there is an inherent scaling limitation for using pairwise strategies on large data sets, since the number of pairs grows with the square of the number of items. Regarding the visualisation approach, we note that a key scaling factor is the amount of similarity links within the corpus—i.e., with relatively few similarity links, the solution will scale reasonably well with corpus size, but if similarity links are abundant, scaling will be challenging. A limiting factor of our computational pipeline is that we use a pairwise comparison strategy which scales poorly to really large corpora (i.e., the number of pairs grows with the square of the number of documents). With the current corpus size of approximately 3000 articles, the necessary pre-calculations (which we to a large extent run on Google Colab A100 GPU instances) take approximately 1.5 hours to complete in sequence. This indicates that, if using a higher level of parallelism for the pre-calculations, the strategy should be a viable option for corpus sizes up to around 10,000 documents (with an estimated preparation time of 6 to 8 hours). Finally, we need to point out that the implementation of Simbanex takes advantage of the fact that similarity is relatively scarce within the data set with regard to the current aspects (i.e., most of the article pairs are dissimilar for most of the aspects.) In a scenario where similarity would be more common, our current computational strategy would have to be revised.

As previously stated, it is not our intention to give the impression that our proposed approach is relevant for all problems within the fields of bibliometrics and scientometrics. We have also pointed out that we see it as a method which should be used in combinations with existing analytical methods, rather than on its own. Nevertheless, we believe that our application gives a relevant contribution, and that it provides a novel approach which could hopefully also be applicable to problems beyond the presented scope. As the main direction for our future work, we plan to leverage the insights from Sect “Use case 2—Topic similarity” and elaborate on how the similarity-based approach could be used for topic detection. Another interesting direction would be to see if a more heterogeneous computational pipeline (i.e., not only using embedding technology for the similarity calculations) could augment the quality of the aspect classifications.

## Supporting information

S1 Video The supporting video describes how the user interface of Simbanex works and how the interactions can be used for different analysis scenarios. (mp4)
